# Supplementary figures and images for: A new Desmodesmus sp. from the Tibetan Yamdrok Lake
Source: PLoS One. 2022 Oct 7;17(10):e0275799. doi: 10.1371/journal.pone.0275799 (PMC9544026; doi:10.1371/journal.pone.0275799)

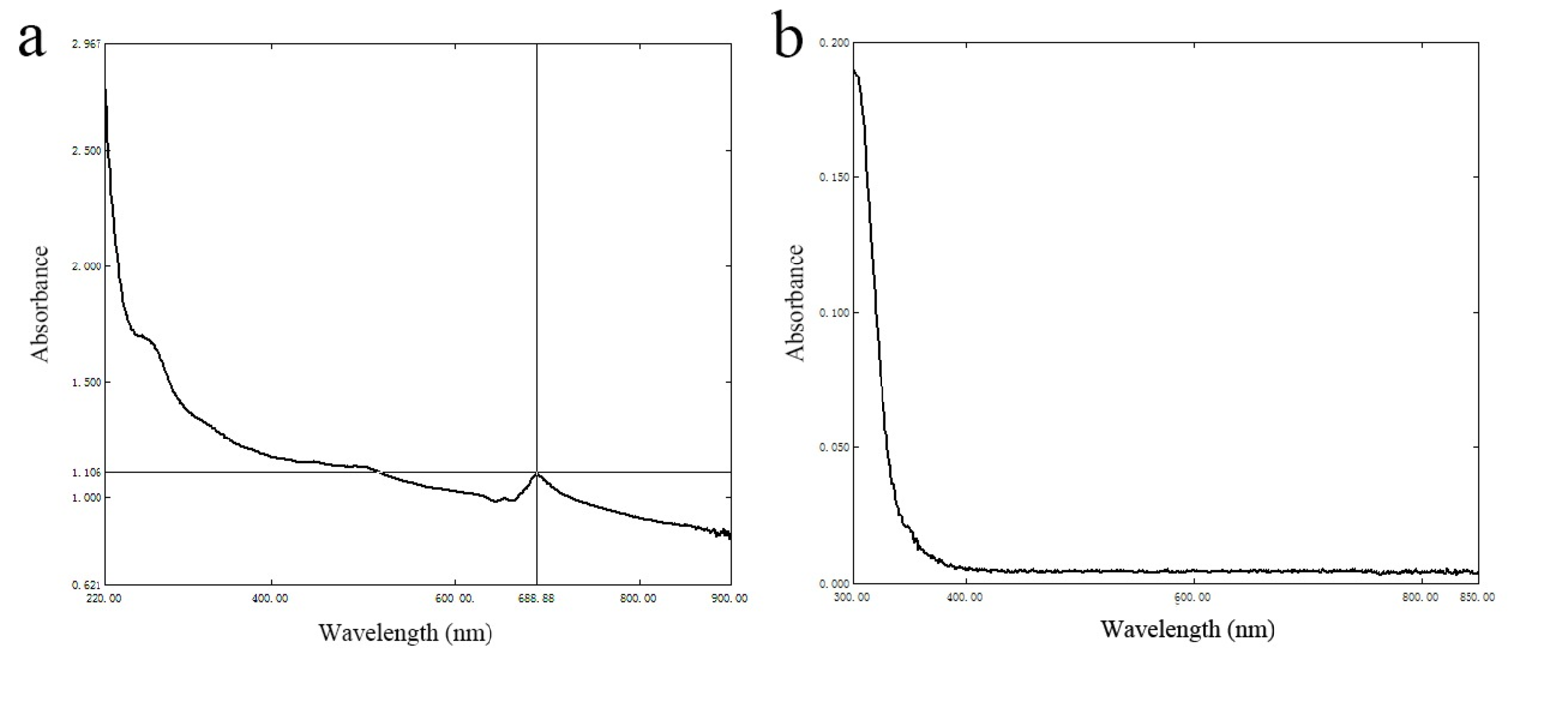

Supplement: S1 Fig — (a) Full wavelength scanning spectrogram of Desmodesmus sp. (b) Full wavelength scanning spectrum of background solution. (TIF) [file pone.0275799.s002.tif]

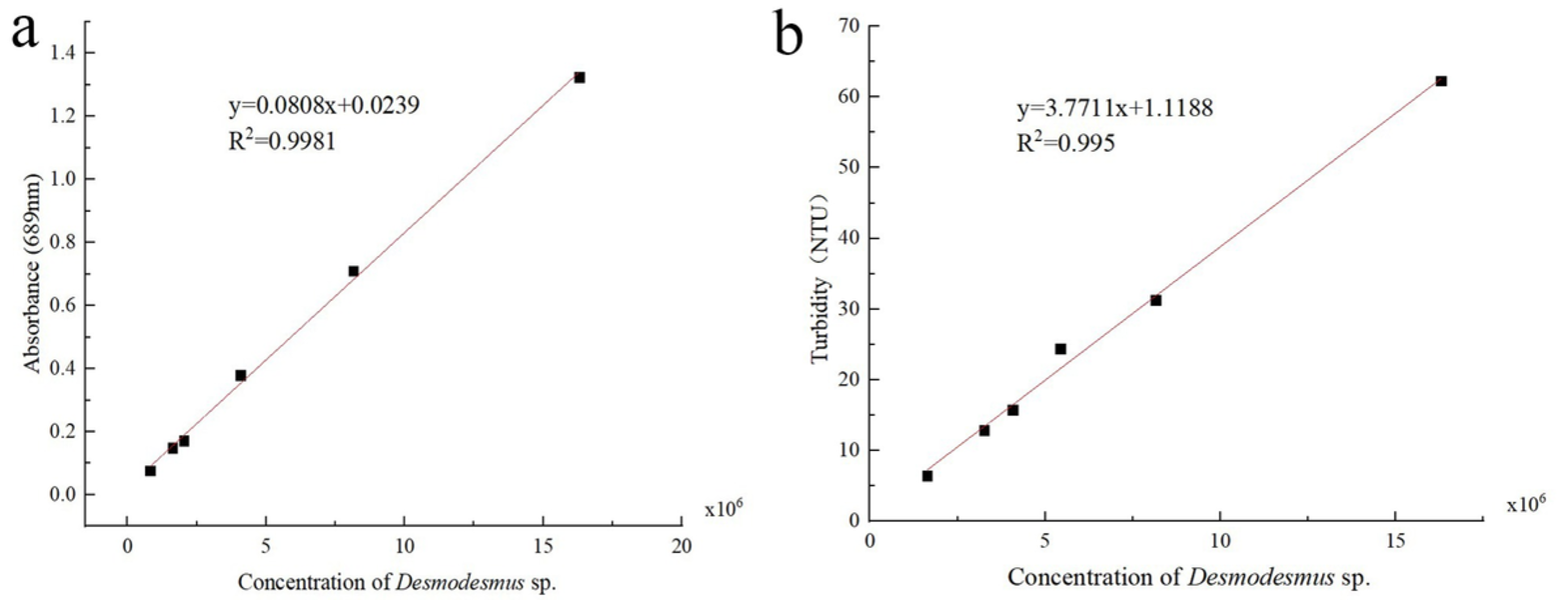

Supplement: S2 Fig — (a) Relationship between microalgae concentration and absorbance of culture solution. (b) Relationship between microalgae concentration and turbidity of culture solution. (TIF) [file pone.0275799.s003.tif]
